# Serum *N*-glycosylation is altered in Nephropathic Cystinosis

**DOI:** 10.1093/glycob/cwaf047

**Published:** 2025-08-22

**Authors:** Andreea Cislaru, Radka Saldova, Alessandra Heggenstaller, Peter A Nigrovic, Emily Harlin, Gordon Greville, Rafael De Andrade Moral, Daniel Bojar, Atif Awan, Róisín O’Flaherty

**Affiliations:** Department of Chemistry, Maynooth University, Maynooth, Co. Kildare W23 F2H6, Ireland; GlycoScience Group, National Institute for Bioprocessing Research and Training (NIBRT), Fosters Avenue, Mount Merrion, Blackrock, Co. Dublin A94X099, Ireland; UCD School of Medicine, College of Health and Agricultural Science (CHAS), University College Dublin (UCD), Dublin 4 D04 V1W8, Ireland; CÚRAM, Science Foundation Ireland Research Centre for Medical Devices, Biomedical Sciences, University of Galway, Co. Galway H91 W2TY, Ireland; Department of Paediatric Nephrology, Children’s Health Ireland (CHI) at Temple Street, Temple Street, Dublin 1 D01 XD99, Ireland; Division of Immunology, Boston Children’s Hospital, Harvard Medical School, Boston, MA 02115, United States; Division of Rheumatology, Inflammation, and Immunity, Brigham and Women’s Hospital, Harvard Medical School, Boston, MA 02115, United States; Department of Chemistry, Maynooth University, Maynooth, Co. Kildare W23 F2H6, Ireland; Department of Biology, Maynooth University, Maynooth, Co. Kildare W23 F2H6, Ireland; Department of Mathematics and Statistics, Maynooth University, Maynooth, Co. Kildare W23 F2H6, Ireland; Wallenberg Centre for Molecular and Translational Medicine, Department of Chemistry and Molecular Biology, University of Gothenburg, Gothenburg 41390, Sweden; Department of Paediatric Nephrology, Children’s Health Ireland (CHI) at Temple Street, Temple Street, Dublin 1 D01 XD99, Ireland; Department of Chemistry, Maynooth University, Maynooth, Co. Kildare W23 F2H6, Ireland; CÚRAM, Science Foundation Ireland Research Centre for Medical Devices, Biomedical Sciences, University of Galway, Co. Galway H91 W2TY, Ireland; Kathleen Lonsdale Institute for Human Health Research, Maynooth University, Maynooth, Co. Kildare W23 F2H6, Ireland

**Keywords:** IgG glycoprotein, lysosomal storage disease, *N*-glycosylation, Nephropathic Cystinosis, serum

## Abstract

Changes in glycosylation can serve as markers for rare genetic disorders, including lysosomal storage diseases (LSDs). Nephropathic Cystinosis (NC), caused by mutations in the CTNS gene, is characterised by cystine accumulation in lysosomes due to dysfunctional cystinosin, a heavily *N*-glycosylated lysosomal transporter. We analysed total serum and IgG *N*-glycosylation using hydrophilic interaction ultra performance liquid chromatography (HILIC-UPLC) to explore the diagnostic biomarker capabilities and their pathophysiological relevance in NC. In this double-blind study (*n* = 12), we examined *N*-glycosylation of total serum and serum IgG from Irish participants with and without NC. Dimensionality reduction methods were used applying their glycan data to predict NC status, yet only modest predictive power was observed (66.6% for serum and 50% for IgG *N*-glycosylation). However, upon unblinding the data, we identified significant differences in specific serum *N*-glycosylation in NC, particularly in sialylation. These findings provide the first evidence that serum *N*-glycosylation is altered in NC. These changes may indicate disease-associated systemic alteration including dysregulation in *N-*glycosylation pathway. It provides justification for the need for a larger validation study and invites further exploration of its role in NC pathophysiology. We provide key recommendations for age stratification for studying serum, plasma and IgG *N*-glycans in juvenile cohorts as they display unique profiles compared to adult populations, an important consideration for all juvenile studies, even beyond the scope of rare diseases.

## Introduction

Degradation and turnover of glycans takes place in the lysosome. It is a crucial organelle that performs cell signalling and maintains cellular harmony by digesting and recycling cell components (Platt et al. 2018). Lysosomal storage diseases (LSDs) are characterised by the accumulation of several macromolecules resulting in abnormalities of autophagy, endocytosis, and inflammation (Boustany 2013). Nephropathic Cystinosis (NC), which often manifests in early infancy, is caused by mutations in the CTNS gene and results in disruption of efficient transport of the amino acid cystine out of lysosomes. This leads to accumulation of cystine as crystals within these organelles, causing cellular and tissue damage; with kidneys and eyes are most affected (Jamalpoor et al. 2021).

Several inflammatory pathways are activated in NC-for example, cell apoptosis is enhanced in cystinotic cells (tissues exhibit proximal tubular epithelial cells (PTECs), fibroblasts and podocytes) and there are noticeable tissue fibrosis and autophagy irregularities (Park et al. 2006; Elmonem et al. 2022). Therefore, directing therapeutic efforts towards monitoring and mitigating inflammation may be a promising strategy. The primary therapy for managing NC, cysteamine, reduces cystine accumulation in cells and can delay kidney dysfunction, yet it does not reverse the cystinotic phenotypes including Glomerulonephritis or Renal Fanconi Syndrome failure (Hauglustaine et al. 1976; Cherqui and Courtoy 2017). Clinical trials are exploring new therapies to address these pathologies phenotypes and provide alternative treatment options. Notably, a stem cell-based approach combined with gene therapy yielded promising findings with Phase 1/11 completed (NCT03897361) (Harrison et al. 2013; Cherqui and Courtoy 2017) and a longitudinal study is ongoing (NCT05146830) (Kido et al. 2023). These advancements have highlighted the need to analyse inflammation*-*related signals and pathways to better understand NC pathophysiology and optimise treatments. Serum and IgG *N*-glycosylation, sensitive to inflammation, could serve as a useful tool. Changes in *N*-glycosylation patterns, including altered galactosylation and sialylation, are often associated with chronic inflammation (Jasmin et al. 2016; Zhou et al. 2021). Given the inflammatory nature of NC, monitoring these glycosylation changes in serum and IgG may provide insights into systemic inflammatory responses. Additionally, these glycosylation patterns could serve as biomarkers to track residual inflammation not addressed by cystine-depleting therapies, facilitating a more comprehensive approach to managing NC.

Glycans are dynamic regulators of the immune response, acting as complex carbohydrate structures on the surfaces of cells and molecules that play a critical role in modulating immune system function. Over 50 different LSDs have been reported to date (Freeze et al. 2022), and at least 60% of these have known defects in glycoproteins, glycolipids and glycosaminoglycan (GAG) degradation. A selection of LSDs with documented alterations and affected immune molecules or disruptions in glycan catabolism is summarised in Table 1.

**Table 1:** LSDs with alterations in immunoglobulin glycoproteins or defects in glycolipid/glycoprotein/glycosaminoglycan degradation.

Lysosomal Storage Disease	Primary Deficiency	Substrate Accumulation	Altered Immune molecules	Affected Immunoglobulin	Elevated Glycosylated Serum Proteins
Gaucher disease (Pratt et al. 1968, Allen et al. 1997, Hollak et al. 1997, Balreira et al. 2005, Pandey and Grabowski 2013)	Acid β-Glucosidase	Glucosylceramide (GlcCer), Glucosylshpingosine (GS)	IL-1, IL-6, IL-8, sCD-14, M-CSF, TNF-α, CD1d, MHC II	Serum IgG, IgA and IgM	C-reactive protein (CRP) (Rogowski et al. 2005) Chitotriosidase (Boven et al. 2004)
Fabry disease (De Francesco et al. 2013)	α-Galactosidase A	Glycosphinolipids, Globotriaosylceramide (Gb3)	IL-1β, IL-6, TNF-α	Unknown	C-reactive protein (CRP) (Rogowski et al. 2005) Lyso-Gb3 (Globotriaosylsphingosine) (Nowak et al. 2022)
Farber disease (Alayoubi et al. 2013)	Lysosomal Acid Ceramidase (ACDase)	Ceramide (Cer)	MCP-1	Unknown	Ceramide C26 (isoform 1 elevated) (Cozma et al. 2017)
Cystinosis (Wilmer et al. 2008a, Prencipe et al. 2014)	Cystinosin	Cystine (Cys)	IL-1β, IL-6, IL-18, TNF-α	Urine IgG (Wilmer et al. 2008a)	Chitotriosidase (Boven et al. 2004)
α-Mannosidosis (Malm et al. 2000)	α-D-Mannosidase	Mannose-containing oligosaccharides	CD11b, CD16	Serum IgG	Insulin-like growth factor 1 (IGF-1) (Jaeggi-Groisman et al. 2005)
Hunter syndrome (Sousa Martins et al. 2022, Żuber et al. 2023)	Iduronic acid-2-sulfatase (IDS)	Dermatan Sulfate (DS) and Heparin Sulfate (HS)	-	Plasma IgG	Heparan sulfate proteoglycans, Dermatan sulfate proteoglycans (de Ruijter et al. 2012)

IL-1, interleukin-1; IL-6, interleukin-6; IL-8, interleukin-8; sCD-14, soluble cluster of differentiation 14; M-CSF, macrophage-colony stimulating factor; TNF-α, tumor necrosis factor-alpha; CD1d, cluster of differentiation 1; MHC II, major histocompatibility complex II; IL-1β, interleukin-1β; MCP-1, monocyte chemoattractant protein 1; IL-18, interleukin-18; CD11b, cluster of differentiation 11b; CD16; cluster of differentiation 16.

In NC, urine IgG titres, and levels of IL-1β, IL-6, IL-18, TNF-α, C-reactive protein, as well as other proteins were found to be significantly elevated (Pratt et al. 1968; Allen et al. 1997; Hollak et al. 1997; Malm et al. 2000; Boven et al. 2004; Balreira et al. 2005; Jaeggi-Groisman et al. 2005; Rogowski et al. 2005; Wilmer et al. 2008a; de Ruijter et al. 2012; Alayoubi et al. 2013; De Francesco et al. 2013; Pandey and Grabowski 2013; Prencipe et al. 2014; Cozma et al. 2017; Nowak et al. 2022; Sousa Martins et al. 2022; Żuber et al. 2023). Additionally, in cystinotic animals a critical link was found between cystinosin and galectin-3 in inflammation (Lobry et al. 2019). Galectin-3 is a member of the galectin family characterised by their ability to bind specifically to β-galactosides on glycoproteins and glycolipids. Possibly most relevant to our study, Nevo and colleagues analysed the ΔITILELP mutation in the *CTNS* gene, which is associated with the juvenile form of cystinosis (Nevo et al. 2017).This mutation disrupts the N66 glycosylation site on cystinosin, leading to impaired glycan maturation. As a result, the protein retains oligomannose glycan structures and undergoes misfolding in the endoplasmic reticulum (ER). This misfolding triggers accelerated degradation in lysosomes and leads to reduced protein stability, contributing to the disease phenotype (Nevo et al. 2017). The authors concluded that the high turnover of ΔITILELP, because of its immature glycosylation state together with low transport activity, might be responsible for the phenotype observed in some patients who carry this mutation heterozygously, together with the 57-kb deletion.

**Figure 1:**
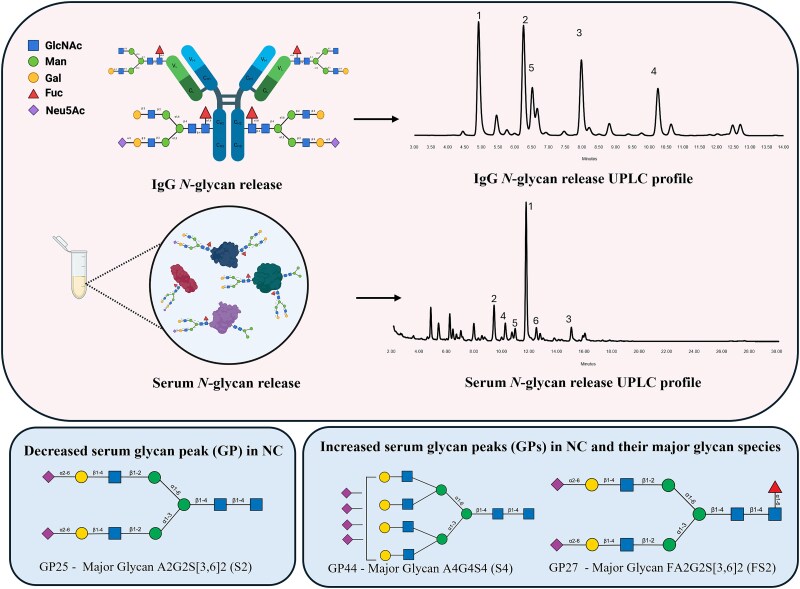
IgG from human serum and total serum *N*-glycans were analysed on HILIC-UPLC. The major peaks are numbered in order of their relative abundance (% area). For IgG the major glycans are: (1) F[6]A2, (2) F[6]A2[6]G[4]1, (3) F[6]A2G[4]2 (4) F[6]A2G[4]2S1, and (5) F[6]A2[3]G[4] (Stöckmann et al. 2015; O'Flaherty et al. 2019). The major glycans for serum are: (1) A2G2S[3,6]2, (2) A2G2S[6]1, (3) A4G4S[3,3,3,6]4, (4) FA2BG2[6]1, (5) A2G2S[3,6]2, and (6) FA2G2S[3,6]2 (Saldova et al. 2014).

Taken together, and given that both innate and adaptive immunity is highly glycan dependent (e.g. proinflammatory cytokines are responsible for affecting levels of many glycosyltransferases and substrate expression pathways) (van Kooyk and Rabinovich 2008; Radovani and Gudelj 2022), we hypothesised that *N*-glycosylation may be differentially abundant in NC. We conducted a pilot study involving female and male juveniles from participants with and without NC (termed the Irish cohort, *n* = 12, aged 2–14 years). This study aimed to compare (a) their total serum *N*-glycosylation and (b) their serum IgG *N*-glycosylation profiles (Fig. 1). Additionally, to enhance the robustness of our findings, the IgG *N*-glycan profiles were compared to those of an additional control cohort (termed the Boston cohort, *n* = 81).

## Results

### Cluster analysis of serum and IgG *N*-glycosylation for discrimination of Nephropathic Cystinosis

To commence the study, a double-blinded investigation was undertaken, involving the *N*-glycoprofiling of total serum and serum IgG from individuals (males and females) both with and without NC (*n* = 12, pseudonyms CY1-CY12, aged 2–14 yr). Experimental approaches using a previously established workflow for serum *N*-glycome analysis (Saldova et al. 2014) and an adaptation of a previously established approach for serum IgG *N*-glycans (O'Flaherty et al. 2019) were followed. Glycans were visualised using HILIC-UPLC and integration of the chromatograms into individual glycan peaks (called GPs, each peak containing one or more glycans) allowed us to generate glycan data. Glycan traits were also calculated from these individual GPs and subsequently these GPs and glycan traits were then harnessed in statistical models to explore the possibility of unequivocally differentiating between the two groups by detecting changes in their total serum and/or IgG *N*-glycosylation data or patterns (glycan peaks and derived glycan traits).

Firstly, we examined the serum *N-*glycosylation for the Irish cohort (*n* = 12, with/without NC) using an already established protocol (Saldova et al. 2014). Briefly, this involved the denaturation and alkylation of sera glycoproteins, followed by the treatment with PNGase F, and fluorescent labelling with 2-aminobenzamide (2-AB). Simultaneously, an IgG *N*-glycan analysis experimental workflow for the Irish cohort was followed which involved affinity purification of IgG from human serum, glycan preparation with denaturation and alkylation, enzymatic release of *N*-glycans using PNGase F, and subsequent fluorescent labelling with 6-aminoquinolyl-*N*-hydroxysuccinimidyl carbamate (AQC), adapted from (Stöckmann et al. 2015). Visualisation and quantification of serum and IgG released *N*-glycans was achieved through hydrophilic interaction ultra performance liquid chromatography (HILIC-UPLC). The resulting serum and IgG *N*-glycoprofiles were integrated into 46 and 23 glycan peaks respectively (called GPs; Table S1 and S2). Derived glycan traits, encompassing the relative levels of neutral, monosylated, galactosylation, sialylated, antennary, and fucosylation (core or outer arm fucose), were summated using an adaptation of previously reported calculations (Table S5.1 and S5.2). This comprehensive approach allowed for a systematic examination of serum and IgG *N*-glycosylation patterns, facilitating the identification of potential discriminatory features between individuals with and without NC within the juvenile cohort.

A separate control juvenile Boston plasma cohort (*n* = 81) was introduced as an additional reference plasma cohort (distinctly different to serum but comprised of many of the same *N*-glycans in adult populations) in an effort to identify the NC (Cheng et al. 2020). In brief, the experimental workflow was as follows: affinity purification of IgG from plasma, involving glycan preparation with denaturation, alkylation, and enzymatic release of *N*-glycans using PNGase F, and subsequent fluorescent labelling with AQC. As before, visualisation and quantification were achieved through HILIC-UPLC (Table S4). Importantly, there were distinct differences with respect to population type and sample preparation between these cohorts. However, owing to the limited data available for juveniles in the literature, the authors decided to expand the data set. For example, the IgG *N*-glycosylation profiles in sera and plasma differ subtly in adults (Amez Martín et al. 2021). As such, comparison between the two groups was done in a cautious manner.

**Figure 2:**
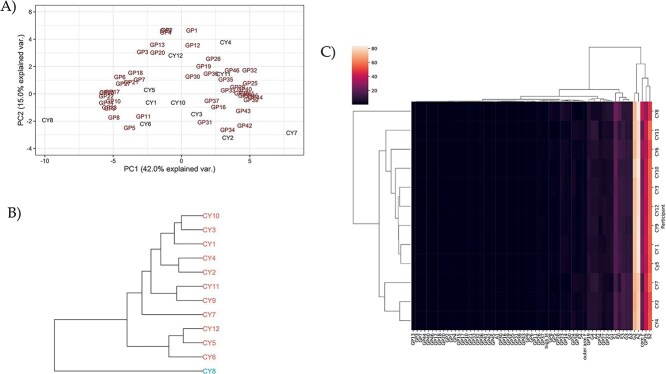
Multivariate analysis of serum *N*-glycosylation for prediction of Nephropathic Cystinosis (NC). A) PCA biplot generated using the 46 GPs, includes the Irish cohort only. B) HC grouping of participants (CY1-CY12), includes the Irish cohort only. C) Heatmap shows the relationship amongst the 46 GPs, IgG titres, and derived glycan traits (Table S1), includes the Irish cohort only.

**Figure 3:**
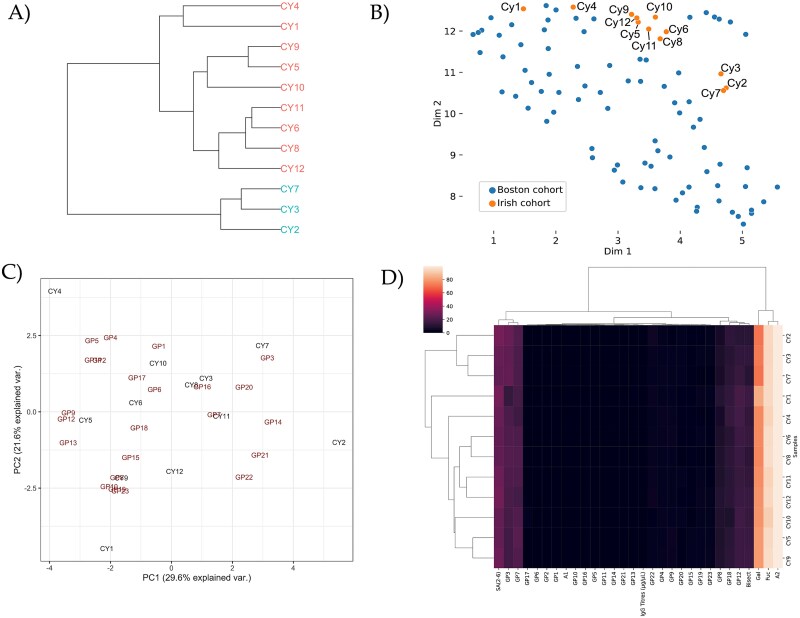
Multivariate analysis of IgG *N*-glycosylation for prediction of Nephropathic Cystinosis (NC). A) HC grouping of participants (CY1-CY12), includes the Irish cohort only. B) UMAP encompasses the Irish cohort (*n* = 12) in orange (annotated with CY1- CY12) and the separate control cohort, the Boston cohort (*n* = 81) in blue (not annotated), according to their GPs. C) PCA biplot generated from IgG *N*-glycan workflow using the 23 GPs, includes the Irish cohort only. D) Heatmap shows the relationship between the 23 GPs, IgG titres, and derived glycan traits (Table S2), includes the Irish cohort only.

In the blinded cohort (*n* = 12), we sought to identify clusters/groupings of participants using statistical models to categorise the NC participants, keeping in mind their small sample size and mixed gender. We hypothesised that a single cluster for those with NC might materialise to set them apart. With that in mind, the total serum and IgG *N*-glycan data were probed separately (Fig. 2 and 3, respectively). The generated GPs, derived glycan traits, and IgG titres were analysed by principal component analysis (PCA) (Cucak et al. 2021), uniform manifold approximation and projection (UMAP) (Bojar et al. 2021), hierarchical clustering (HC), and heat maps (Thomès et al. 2021, Thomès et al. 2023). In the UMAP, the Boston control cohort was incorporated as a reference control cohort (Fig. 3, Table S4) (Cheng et al. 2020).

Thereafter, in this double-blinded study, we attempted to predict NC status using serum *N*-glycan data and IgG *N*-glycan data, bearing in mind that cohort size was a major limitation. Viewing at the serum *N*-glycan data, clustering was observed, and participants were grouped as follows: group 1 (CY2 and CY4), group 2 (CY9 and 11), group 3 (CY5 and CY12), and group 4 (CY3 and CY10) based on the HC (Fig. 2B). A separate group was identified by PCA (Fig. 2A), namely group 5 (CY1, CY5, and CY10). Separately, the heatmap (Fig. 2C) identified the following clustering patterns and participants were grouped accordingly: group 6 (CY4, CY2, and CY5), and group 7 (CY1, CY3, CY5, CY9, CY10, and CY12). Considering that participants CY2, CY3, and CY7, participants CY1 and CY4, and participants CY1 and CY12 (Fig. S1) appeared to be clustered separately, we predicted that they formed the same groups. Taken together, we tentatively predicted that participants labelled CY1, CY5, CY6, CY9, CY11, CY12 form one group and CY2, CY3, CY4, CY7, CY8, and CY10 the other, based on serum *N*-glycosylation results (Table 2).

**Table 2:** Predictions of NC status of blinded data (*n* = 6 NC and *n* = 6 controls) of serum and IgG *N*-glycans based on PCA, HC, and heatmap (Fig. 2 and 3) and unblinded results. Unblinding revealed a 67% and 50% accuracy for prediction for NC status.

**Sample (Sex)**	**Prediction (Serum)**	**Prediction (IgG)**	**Unblinded**
CY1 (F)	Cystinosis	Controls	Cystinosis
CY2 (F)	Controls	Controls	Controls
CY3 (M)	Controls	Controls	Cystinosis
CY4 (F)	Controls	Controls	Controls
CY5 (M)	Cystinosis	Cystinosis	Cystinosis
CY6 (F)	Cystinosis	Cystinosis	Controls
CY7 (M)	Controls	Controls	Cystinosis
CY8 (M)	Controls	Cystinosis	Controls
CY9 (M)	Cystinosis	Cystinosis	Cystinosis
CY10 (F)	Controls	Cystinosis	Controls
CY11 (M)	Cystinosis	Cystinosis	Cystinosis
CY12 (M)	Cystinosis	Controls	Controls

The same approach was taken for IgG *N*-glycan data, participants were grouped as follows: group 1 (CY3, CY8, and CY10), group 2 (CY9 and CY12) according to the PCA biplot (Fig. 3C). A separate group 3 (CY2, CY3, and CY7) was established based on clustered in the HC, UMAP, and heatmap, possibly driven by changes in galactosylation (Fig. 3A, 3B and 3D). Participants CY1 and CY4 also clustered in HC and UMAP. Based on the IgG *N*-glycosylation, we predicted CY1, CY2, CY3, CY4, CY7, and CY12 formed one group and CY5, CY6, CY8, CY9, CY10, and CY11 formed another group (Table 2). Notably, there was not much overlap between the two groups and as such we did not have much confidence that we could accurately predict NC in a blinded manner. Following unblinding of the data, based on the serum and IgG *N*-glycan analysis, 66.6% and 50%, respectively, of participants were correctly identified based on their NC status (Table 2).

### Effects of age and sex on serum and IgG *N*-glycosylation in the Irish cohort

**Figure 4:**
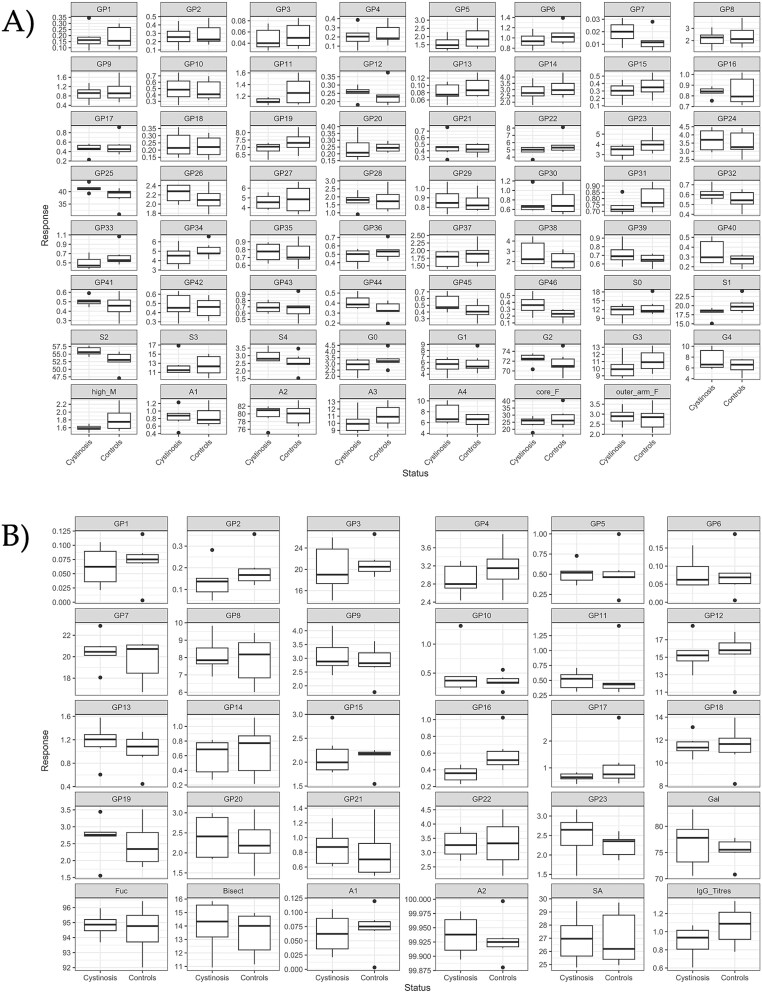
Following the unblinding of the Irish cohort (*n* = 12, males and females): A) boxplot analysis of serum *N*-glycosylation data for all 46 GPs and traits for controls (*n* = 6) and NC participants (*n* = 6). B) Boxplot analysis of IgG *N*-glycosylation data for 23 GPs, traits, and IgG titres for controls (*n* = 6) and NC participants (*n* = 6).

**Figure 5:**
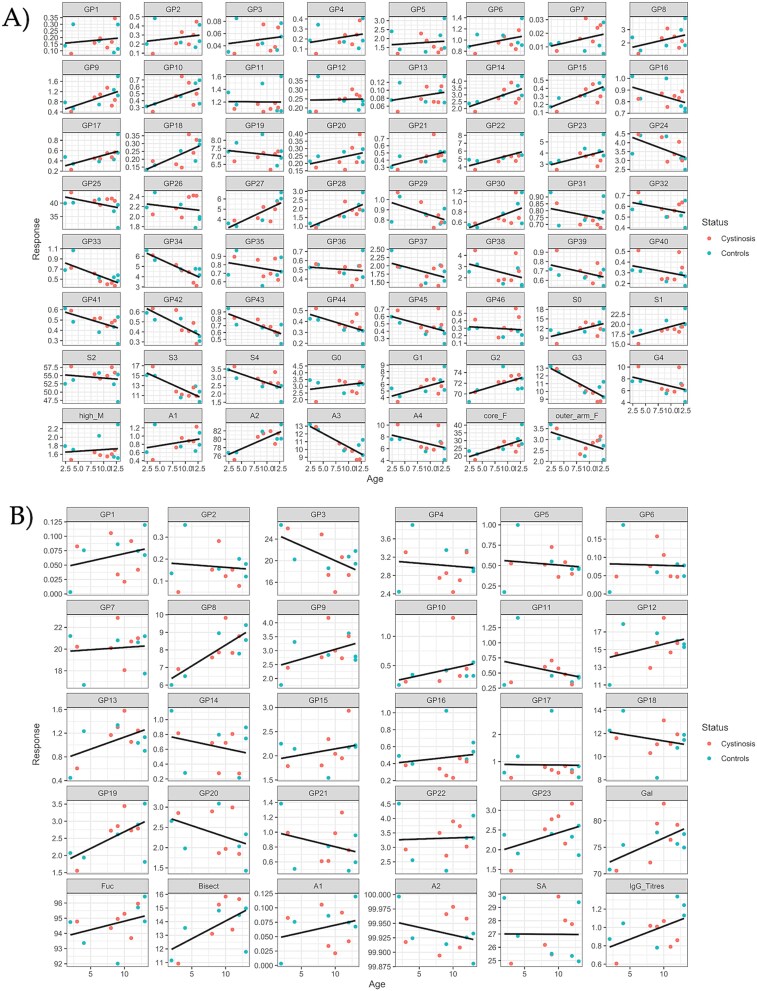
Following unblinding of the Irish cohort a) linear regression analysis for 46 GPs and traits for the controls (*n* = 6) and the NC participants (*n* = 6) with respect to age. B) Linear regression analysis of IgG *N*-glycosylation data for 23 GPs, traits, and IgG titres for controls (*n* = 6) and NC participants (*n* = 6) with respect to age.

Unblinding of the Irish cohort (*n* = 12) allowed for in-depth analysis of the specific role of glycans in this cohort. Glycosylation data, namely glycan peaks (GPs, note that each GP can contain one/more glycan), traits and IgG titres were analysed using boxplots and linear regression model analyses (Fig. 4 and Fig. 5, Fig. S2 and S3). *P*-values were calculated using sequential ANOVA (type-I sums of squares) F-tests to assess the significance of the effects on sex, age, and NC status (Table 3 and 4).

**Table 3:** Irish cohort (*n* = 12) *P*-values carried out using multivariate ANOVA analysis of serum *N*-glycosylation parameters. F-tests for the effects of sex, age (corrected for sex), and NC status (corrected for age and sex) for each GP and traits were performed. *P*-values < 0.05 (statistically significant at a 5% level are in bold).

GP’s & Traits	Sex	Age	Status	GP’s & Traits	Sex	Age	Status
GP1	0.634	0.770	0.699	GP33	0.131	**0.009**	0.108
GP2	0.660	0.657	0.606	GP34	**0.033**	**0.001**	0.125
GP3	0.460	0.724	0.337	GP35	0.375	0.222	0.673
GP4	0.493	0.528	0.447	GP36	0.495	0.458	0.085
GP5	0.922	0.736	0.249	GP37	0.156	0.256	0.798
GP6	0.804	0.252	0.269	GP38	0.832	0.162	0.387
GP7	0.143	0.487	0.453	GP39	0.185	**0.037**	0.691
GP8	0.874	0.189	0.630	GP40	0.437	0.179	0.386
GP9	0.797	**0.036**	0.573	GP41	0.769	**0.028**	0.399
GP10	0.909	0.058	0.544	GP42	0.341	**0.005**	0.536
GP11	0.654	0.915	0.148	GP43	0.349	**0.006**	0.595
GP12	0.646	0.781	0.465	GP44	0.775	**0.011**	**0.035**
GP13	0.936	0.399	0.377	GP45	0.977	0.063	0.145
GP14	0.619	**0.014**	0.718	GP46	0.482	0.517	0.112
GP15	0.818	**0.006**	0.439	S0	0.966	0.074	0.386
GP16	0.125	0.220	0.516	S1	0.999	**0.01**	**0.031**
GP17	0.356	**0.057**	0.246	S2	0.593	0.436	0.084
GP18	0.589	**0.024**	0.666	S3	0.390	**0.004**	0.866
GP19	0.702	0.571	0.222	S4	0.855	**0.006**	0.087
GP20	0.588	0.138	0.724	G0	0.684	0.445	0.125
GP21	0.217	**0.009**	0.056	G1	0.893	0.066	0.728
GP22	0.700	**0.027**	0.082	G2	0.654	**0.047**	0.258
GP23	0.832	**0.049**	0.134	G3	0.074	**0.001**	0.091
GP24	0.534	0.083	0.465	G4	0.744	0.114	0.336
GP25	0.781	0.102	**0.039**	high_M	0.797	0.614	0.138
GP26	0.584	0.634	0.190	A1	0.838	0.408	0.940
GP27	**0.004**	**0.001**	**0.019**	A2	0.544	**0.005**	0.880
GP28	0.197	**0.007**	0.537	A3	0.074	**0.001**	0.091
GP29	0.327	**0.026**	0.602	A4	0.744	0.114	0.336
GP30	0.405	0.094	0.586	core_F	0.524	**0.019**	0.182
GP31	0.963	0.255	0.101	outer_arm_F	0.463	0.093	0.530
GP32	0.548	0.143	0.470				

**Table 4:** Irish cohort (*n* = 12) *P*-values carried out using multivariate ANOVA analysis of IgG *N*-glycosylation parameters. F-tests are carried out for the effects of sex, age (corrected for sex), and NC status (corrected for age and sex) for each GP, trait and IgG titres were performed. *P*-values *P* < 0.05 (statistically significant at a 5% level) and *P*-values <0.10 (marginally significant) are in bold.

GPs/Traits/Titres	Sex	Age	Status
GP1	0.249	0.567	0.298
GP2	0.384	0.976	0.559
GP3	0.547	**0.016**	**0.092**
GP4	0.652	0.857	0.413
GP5	0.939	0.681	0.847
GP6	0.647	0.970	0.685
GP7	0.372	0.978	0.804
GP8	0.490	**0.003**	0.296
GP9	0.801	0.187	0.344
GP10	0.303	0.108	**0. 057**
GP11	0.475	0.514	0.953
GP12	0.341	0.131	0.400
GP13	0.650	**0.041**	**0.081**
GP14	0.906	0.411	0.450
GP15	0.544	0.532	0.956
GP16	0.478	0.405	**0.081**
GP17	0.226	0.699	0.664
GP18	0.743	0.514	0.849
GP19	0.931	**0.023**	0.167
GP20	0.935	0.239	0.759
GP21	0.973	0.385	0.739
GP22	0.941	0.915	0.914
GP23	0.975	0.132	0.168
Gal	0.594	**0.012**	**0.059**
Fuc	0.337	0.421	0.932
Bisect	0.617	**0.016**	**0.096**
A1	0.249	0.567	0.298
A2	0.248	0.567	0.297
SA	0.539	0.849	0.656
IgG_Titres	0.507	**0.038**	0.278

For the small sample set, when comparing the NC (*n* = 6) and control sera samples (*n* = 6), the *P*-values generated for serum *N*-glycans revealed that only glycan peaks GP27 (*P* = 0.04) and GP34 (*P* = 0.033) were significant with respect to sex (Table 3). No statistical significance was observed between the males and females with respect to their IgG *N-*glycans (Table 4, Fig. S3). Taken together, this is broadly in agreement with literature, where sex differences are less distinct in children compared to adults and present mainly during puberty (Pučić et al. 2012; Cheng et al. 2020). The following serum GPs which were found to be significant with respect to ageing: GP9 (*P* = 0.036), GP14 (*P* = 0.014), GP15 (*P* = 0.006), GP17 (*P* = 0.057), GP18 (*P* = 0.024), GP21 (*P* = 0.009), GP22 (*P* = 0.027), GP23 (*P* = 0.049), GP27 (*P* = 0.001), GP28 (*P* = 0.007), GP33 (*P* = 0.009) and GP34 (*P* = 0.001) are increasing with respect to increasing age (Fig. 5A). The following decreased with respects to increasing age: GP29 (*P* = 0.026), GP39 (*P* = 0.037), GP41 (*P* = 0.028), GP42 (*P* = 0.005), GP43 (*P* = 0.006), and GP44 (*P* = 0.011), (Fig. 5A). The following glycan traits increased: S1 (*P* = 0.010), G2 (*P* = 0.047), A2 (*P* = 0.005) and core fucose (*P* = 0.019); while S3 (*P* = 0.004), S4 (*P* = 0.006), G3 (*P* = 0.001), and A3 (*P* = 0.001) decreased with respect to increase in age (Fig. 5A). Tri- and tetra-sialylation decreased, with an increase in mono-sialylation, while tri-galactosylated species decreased and core-fucosylation and biantennary glycans increased with increasing age, as shown in the regression plot (Fig. 5A). With widespread interest in the concept of glycans changing with age including works by our team and others (Lado-Baleato et al. 2024), and commercial successes too, e.g. Glycan Age, this study also adds further to the narrative by providing an insight into juvenile ageing (Lado-Baleato et al. 2024; Šimunić-Briški et al. 2024).

With the limited research on serum *N*-glycosylation in juvenile cohorts, we compared our serum cohort to a plasma cohort with the working knowledge that plasma and serum *N*-glycosylation have major overlap in glycans, and have similar trends, where tri- and tetra-sialylated glycans are decreasing with age (Pučić et al. 2012). We observed statistical significance for age (*P* < 0.05, corrected for sex) for seven different IgG glycan features/IgG titre (GP3 (p 0.016), GP8 (*P* = 0.003), GP13 (*P* = 0.041), GP19 (*P* = 0.023), Gal (*P* = 0.012), Bisect (*P* = 0.016), and IgG titre (*P* = 0.038). Using linear regression analysis, we observed increases in glycan peaks GP8, GP13, and GP19, and glycan traits Gal, Bisect, and IgG titre with respect to age and decreases for GP3 (Fig. 5B). These findings were unsurprising, considering we reported significant changes in glycosylation across age ranges in a juvenile population previously (Cheng et al. 2020).

Finally, knowing the effects of sex and age on this cohort, we looked further at the NC status (corrected for age and sex) to try to unlock the role of glycans in NC. Serum *N*-glycans have significant GPs associated with the status of the NC cohort: GP21 (*P* = 0.056) is marginally significant, while GP25 (*P* = 0.039), GP27 (*P* = 0.019), GP44 (*P* = 0.035), and S1 (mono-sialylated glycans, *P* = 0.031) are all significant. GP25 contains a di-sialylated glycan, A2G2S2, as a major glycan that was more abundant in NC than in the control group. GP27 on the other hand contains the major glycan FA2G2S2 and was less abundant in NC compared to the control group. GP44 containing the major glycan FA4G4S4 was more abundant in NC compared to the control group. Lastly, the S1 glycan trait was less variable and less abundant in NC compared to the control group (Fig. 4A).

### Effects of age and sex on IgG *N*-glycosylation in control Boston cohort

As described previously, a control cohort from a population in Boston was used as a reference population. IgG was purified from the Boston plasma samples (*n* = 81, ages 2–14 yrs) using affinity chromatography. The released *N*-glycans were labelled with AQC and glycoanalysed using HILIC-UPLC (described in Materials and Methods section). Generated glycan peaks (GP1-GP23) and biological manifest are presented in Table S4. This cohort served a dual functionality: 1) to predict which participants may/may not have NC in the Irish cohort and 2) to assess and validate the trends relating to age and sex in a large juvenile population. We found that this population was not predictive of the control status in the Irish cohort as the two cohorts were markedly different in the discrimination models (Fig. 3B) and the Boston cohort was not useful in that regard. However, it did serve as a meaningful way to consider the trends in IgG *N*-glycosylation relating to age and sex in a large juvenile population. As such, we classified our population into three groups: juveniles younger than 5 yr old, those between 5 and 10 yr, and those between 10 and 14 yr (Boxplots in Fig. S4.A and Table S6) (Cheng et al. 2020) and compared their IgG *N*-glycosylation.

We subsequently stratified their IgG *N*-glycans (GP1-GP23) by sex (Fig. S4.B and Table S6). Only GP 16 (*P* = 0.029) was found to be significant unlike the Irish cohort (Table 3), where no significance was found with respect to sex. Contrastingly, age was significant across multiple GPs (GP1, GP3, GP4, GP6, GP7, GP8, GP9, GP10, GP12, GP13, GP14, GP16, GP17, GP18, GP20, and GP21, all with *P* < 0.05) for the Boston cohort (Table S6). Additionally, post-hoc multiple comparison analysis (Table S7) shows high variation across age ranges, justifying classification into these age ranges for future studies. This is critical for any studies involving serum and/or IgG *N*-glycosylation, even beyond the current scope of this study.

## Discussion

In this study, we endeavoured to discern individuals afflicted with NC from their control counterparts within a juvenile demographic using serum and IgG *N*-glycosylation. Statistical methodologies and clustering models were applied in a blinded framework. Subsequently, upon unveiling the dataset, we sought to delineate discernible patterns, e.g. glycan peaks/traits that could indicate a role in the disease. Finally, a comparative analysis was conducted by juxtaposing our findings with those of another juvenile cohort, though subtly different (plasma IgG *N*-glycans), to unveil potential correlations and trends across datasets.

The serum *N*-glycosylation has a slightly higher predictive accuracy (66.6%) compared to IgG *N*-glycosylation (50%). These results do not confidently distinguish between the two groups that were predicted, although serum *N*-glycan analysis had a higher prediction rate, suggesting that other serum proteins other than IgG may be contributing to this predictive power. Importantly, the Irish cohort consisting of participants with/without NC clustered differently to the Boston control cohort that was used trying to identify the Irish cohort without NC. As such, the Boston cohort was not useful to predict NC status in this study. These differences were possibly down to small changes in preparation protocols, differences in their heterogeneity but most likely arise from the fact that plasma and serum *N*-glycans, although containing the same glycans, cannot be compared in such a manner. As such, we were not able to predict their status using this cohort with any degree of confidence. Serum and IgG *N*-glycosylation exhibits variability among individuals on a population level in adults and has been studied extensively (Yamada et al. 1997; Vanhooren et al. 2007; Ding et al. 2011; Pučić et al. 2011; Lado-Baleato et al. 2024). However, research on sera glycosylation in juvenile populations is very limited. Until now, no comprehensive characterisation of juvenile human serum *N-*glycosylation of a specific age range (aged 2–14 yr) has been reported in the literature using HILIC-UPLC. Chen et al. and other others have previously looked at plasma or sera *N*-glycosylation with larger age ranges with respect to other metabolic diseases (Chen et al. 2019; Garapati et al. 2024). With our colleagues, we previously characterised juvenile cohorts on a plasma IgG Fc *N*-glycosylation level (Cheng et al. 2020). It was found that there were striking differences compared to adult populations. Importantly, 1) post-menstruation females displayed adult-like IgG *N*-glycosylation and 2) their IgG *N*-glycosylation varied across different age categories. Taking this into consideration, in this pilot study, we selected for a specific age range in our study (aged 2–14 yr) and excluded menarche (refers to the first menstruating cycle) females, however we opted for a mixed-sex cohort as the sample size was small. Importantly, this study provides important reference material for healthy juvenile populations for serum *N*-glycans, serum IgG *N*-glycans (Irish cohort) and plasma IgG *N*-glycans (Boston cohort) for the first time using HILIC-UPLC chromatography. This should inform future glycosylation studies in juveniles.

Overall, these results highlighted three major limitations of our study: 1) the size of the cohort, 2) the uneven distribution of sex, and 3) the age spread (aged 2–14 yr) of participants may have limited the power of prediction. Most importantly, the size of the cohort significantly restricted this pilot study. By design, every juvenile NC participant in Ireland (aged 2–14 yr, excluding menstruating females only) was enrolled in this study in the hope that, if therapeutically relevant, the project could later be expanded to a larger European network of NC participants in a follow-up study.

Furthermore, we were interested to see whether age and sex are confounding factors in our study and other related studies in the future. The effect of NC status (after correcting for age and sex) for IgG glycans was significant at 10% (*P* < 0.10) for six different glycan peaks or features (GP3, GP10, GP13, GP16, Gal, and Bisect). Interestingly, all agalactosylated glycan species (structures lacking galactose, e.g. A2, also called G0) are decreased in the NC cohort (boxplots in Fig. 3C and linear regression 4D, GP1-GP4). The glycan peak GP3 containing the most abundant agalactosylated glycan, FA2, displayed different abundances between the two NC status at a 10% significance level (*P* = 0.092). Additionally, the galactosylated glycans (Gal, *P* = 0.059) and individual GPs associated with galactosylation (GP10 (*P* = 0.057), GP13 (*P* = 0.081), and GP16 (*P* = 0.081)) are all only partially statistically relevant. This is positive news for experimental design studies on rare disease that may need to utilise males and females together where needed, although the authors would still recommend sex separation where possible based on the significant differences observed for some GPs on a serum level. When we correct for sex and compare the two groups for age, age factors are a significant driver for differences with respects to serum and IgG *N*-glycans, mirroring findings in adult populations (Pučić et al. 2012; Lado-Baleato et al. 2024).

Alterations in plasma *N*-glycans have been observed in other metabolic disorders such as Classical Galactosemia (CG); in one study of females aged 2–9 yrs, FA2G1S1 glycans were found to be elevated (Liu et al. 2012), and in this NC study, sialylation is likewise altered. IgG *N*-glycans are also affected in CG and in MAN1B1-CDG, with shifts in the ratios of agalactosylated to mono- or di-galactosylated glycans (e.g. G0/G1 and G0/G2) previously demonstrated (Saldova et al. 2015; Maratha et al. 2016; Treacy et al. 2021). Moreover, knockdown of *Mgat5* (mannoside acetylglucosaminyltransferase 5, responsible for the addition of GlcNAc) in mice has been shown to alter *N*-glycan complexity in a manner correlating with disease severity in Niemann–Pick type C, a neurodegenerative LSD (Cawley et al. 2022). These findings suggest that glycans may also contribute to the pathophysiology of NC, although further studies are warranted.

Glycan dysregulation is not uncommon in LSDs. For example, in Fucosidosis, impaired glycan degradation via FUCA1 leads to the accumulation of glycans and autophagosomes in lysosomes (Baudot et al. 2022). Lysosomal disruption is also seen in NC, where the cystinosin transporter protein that contains several *N*-glycosylation sites has lost function. The glycosylation site is essential for proper folding, and stability, it’s post-translational modification influences cystinosin ability to interact with other lysosomal protein and facilitate their transport (Cherqui and Courtoy 2017). In our NC study, an LSD with distinct pathophysiology showed evident changes in serum *N*-glycosylation between control and NC participant indicating that like other LSDs, NC also has *N*-glycosylation dysregulation. Importantly, the degree of specificity of this dysregulation in NC compared to other autoimmune/inflammatory disorders is yet to be explored. IgG *N*-glycosylation, on the other hand, has not proven to be of significance in discerning between NC and controls, at least in this small cohort, indicating that other serum glycoproteins may be responsible for the *N*-glycosylation changes in serum. Therefore, a more in-depth structural analysis-focusing on specific linkages and glycan epitopes, and glycoprotein specific studies, (O'Flaherty et al. 2019) may distinguish cystinosis-associated glycosylation changes from those seen in other systemic, LSDs or liver-related diseases.

## Conclusion

In a double-blinded pilot study, we aimed to distinguish between Nephropathic Cystinosis (NC) and control participants in a juvenile Irish population (*n* = 12, aged 2–14 yrs, males and females) by comparing their serum and IgG *N*-glycosylation profiles. We were able to correctly predict 67% and 50% of NC participants using serum and IgG *N*-glycans respectively. Following unblinding of the data, when correcting for age and sex, NC status shows significance for several glycan peaks (GP25, GP27, GP44) in the LC chromatogram as well as the S1 (mono-sialylated glycans) glycan derived trait for serum *N*-glycosylation. For IgG *N*-glycosylation, the NC status neared significance for several glycan peaks and traits, most notably those associated with the absence of galactosylation (G0). Overall, these findings strongly suggest that serum glycosylation—particularly sialylation—and possibly IgG *N*-glycosylation are promising biomarkers for diagnosis and for advancing our understanding of metabolic differences in individuals with NC. A larger sample size would increase the statistical confidence in these findings; especially to clarify the role of sialylated glycans and possibly galactosylated glycans in sera *N*-glycome studies. Altogether, this would improve a predictive model for NC status. Based on these insights, we propose expanding the study to a larger cohort, with a focus on carefully considering sex and age. Future research should investigate which serum glycoproteins, if any, underlie these glycosylation changes.

Comparison with a larger plasma control dataset revealed compelling insights about juvenile IgG and serum/plasma *N*-glycosylation and the differences of juvenile glycosylation compared to adult populations. We demonstrated that age plays a significant role in serum, plasma and IgG *N*-glycosylation across healthy juvenile cohorts. Contrastingly, sex was not a significant factor overall. However, some GPs in the serum *N*-glycosylation profiles were also found to be significant with respect to sex stratification. These findings are important for analysing serum, plasma and IgG *N*-glycosylation in future juvenile studies. Our recommendations from this study for juvenile populations are to stratify by sex where possible and to have the following classifications for age: younger than 5 yr old (aged 2–5 yr), those between 5 and 10 yr (aged 5–10 yr), and those between 10 and 14 yr (aged 10–14 yr). Importantly, this paper also presents the first comprehensive study of total IgG *N*-glycans in healthy juvenile plasma samples. Relatedly, using HILIC-UPLC, this study provides important reference data for juvenile serum *N-*glycans, serum IgG *N*-glycans and plasma IgG *N*-glycans for the first time.

## Materials and methods

### Chemicals and equipment

Milli-Q water was used for all buffer preparations and washing steps, generated from Millipore Milli-Q Integral 3 A10 TOC system. Pre-packed 300 μL tip columns with 20 μL of Protein G resin were obtained from Phynexus. 96 Well Plate Greiner, Nunc Plates and Robotic Reservoirs Flat bottom plates were purchased from ThermoFisher Scientific. 2-AB Labelled Dextran (from Waters) and HPLC Vials (0.3 mL PP Short Thread Micro-Vial) were purchased from Apex Scientific. Acetonitrile (MeCN, HPLC Grade) and Methanol (MeOH) were purchased from Honeywell. Dithiothreitol (DTT), normal human serum, sodium dodecyl sulfate (SDS), IgG from human serum, iodoacetamide (IAA), sodium bicarbonate (SBC), sodium Phosphate Dibasic (Na_2_HPO_4_), *N*,*N*,*N*,*N*-tetramethyl-ethylenediamine (TEMED), formic acid, sodium cyanoboro-hydride (NAB_3_CN), acetic acid (AcOH) and dimethyl sulfoxide (DMSO) were all purchased from Sigma-Aldrich. 2-Aminobenzamide (2-AB) was purchased from Alfa Aesar. Sodium chloride (NaCl), Sodium Azide (NaN_3_), Potassium Chloride (KCl), Phosphate Monobasic (NaH_2_PO_4_), Tris-hydrochloride (Tris–HCl) were all were purchased from Fisher. Glycine (CALBIOCHEM), PNGase F (New England BioLabs), 6-aminoquinolyl-*N*-hydroxysuccinimidyl carbamate (Biosynth Carbosynth), Nanoseps Omega 10 KDa Centrifugal Devices (Thermo Life Sciences) and HyperSep-Diol plate 100 mg Well Plate (ThermoFisher Scientific) were used. Whatman 3MM CHR chromatography paper purchased from Cytiva. Ammonium peroxodisuphate (APS) purchased from AnalaR; BDH 100323 W. Protogel (30%) (w/w) acrylamide: 0.8% (w/v) bis-acrylamide stock solution (37.5:1) (Protogel ultrapure protein and sequencing electrophoresis grade, gas stabilised)) was purchased from National Diagnostics, Hessle, Hull, UK, EC-890. Glycan preparation and release was performed on a Hamilton MicroLab Starlet Liquid Handling Station (Hamilton Bonaduz, AG, Switzerland) equipped with a 96-software Venus3 controlled pipettes and an automated heater shaker using Hamilton Robotics Venus Two software. Protein concentrations were measured on a Perkin Elmer UV–Vis Lambda 365 with Perkin Elmer UV WinLab Software. Fluorescent samples were measured using a Waters ACQUITY UPLC H-Class with Empower 3 Operating Software equipped with an ACQUITY UPLC Glycan BEH Amide Column, (130 Å, 1.7 μm, 2.1 mm x 150 mm).

### Subjects

This study was approved by the Ethics Review Committees of Children’s Hospital Ireland (CHI), Temple Street (Dublin, Ireland), Maynooth University (MU) (Kildare, Ireland) and Boston Children’s Hospital (Boston, USA) (see Institutional Review Board Statement). For the Irish Cystinosis cohort, serum samples from six control juvenile participants (hereafter called controls) and six cystinosis patients were used (aged 2–14 yrs). All patients were diagnosed by estimation of white cell cystine levels following genetic confirmation and underwent cysteamine treatment. After allowing the blood to clot for 30–60 min, serum was obtained by centrifugation at 2000 x g for 10 min and were stored at −80 °C until analysis. The samples were blinded by collaborates at CHI, and the pseudonyms were labelled CY1–12 where each sample’s number corresponded to a patient. For the Boston control cohort, plasma from control children (ages 2–14 yrs) was prepared as described previously (Cheng et al. 2020). In short, plasma was obtained by centrifugation at 2000 x g for 10 min and stored at −80 °C prior to analysis. To ensure confidentiality and double blinding, all patient data and samples were assigned a coded pseudonym at the point of collection. Only the nephrologist and clinical coordinator had access to the identifying information. All subsequent sample handling (e.g. serum separation, storage, glycomics analysis) was performed without access to clinical identifiers.

Laboratory personnel involved in the glycan extraction, processing, and analysis were blinded to the disease status of each sample. The NC/control grouping was only unblinded after statistical analysis was complete. This procedure ensured independent data interpretation and reduced the risk of observer bias.

### Serum analysis of *N*-glycans and labelling (2-AB) for Irish cohort

The experimental protocol was described previously (Saldova et al. 2014). All sera samples from the Irish cohort were tested (*n* = 12) as well as pooled human serum (*n* = 3) and were used as technical replicates. Briefly, the following buffers were prepared: TRIS (stacking buffer, 0.5 M, pH 6.6), 10% SDS, TRIS (gel buffer, 1.5 M, pH 8.8), Protogel (30% (w/w) acrylamide: 0.8% (w/w) bis-acrylamide stock solution (37.5: 1) Protogel ultrapure protein and sequencing electrophoresis grade, gas stabilised), 10% APS, sodium bicarbonate (20 mM, pH 7), DTT (0.5 M), IAA (0.1 M) and 1% formic acid. The sera samples (5 mL), 2 mL of sample buffer (62.5 mL of 0.5 M TRIS pH 6.6, 100 mL of 10% SDS, and 337.5 mL water), 2 mL water and 1 mL of 0.5 M DTT were mixed gently and incubated at 65 °C for 15 min. The samples were alkylated by adding 1 mL of 100 mM IAA and incubated in the dark at room temperature for 30 min. The samples were set into gel block by 22.5 mL of 30% (w/w) acrylamide/0.8% (w/v) bis-acrylamide stock solution (37.5:1.0, Protogel, National Diagnostics, Hessle, Hull, UK), 11.25 mL of 1.5 M Tris (pH 8.8), 1 mL of 10% SDS, 1 mL of 10% ammonium peroxodisulfate (APS), and finally 1 mL of *N*,*N*,*N*,*N*-tetramethyl-ethylenediamine (TEMED), mixed, and allowed to set for 15 min and transferred to a – 20 °C for 5 min. The gel was removed and cut into 1 mm^3^ pieces. Each sample was washed with 1 mL MeCN and vortexed for 10 min, then 1 mL 20 mM NaHCO_3_, and vortexed for 10 min (this was repeated x2 times) and finally the samples were dried. *N*-glycans were released by adding 200 mL of 1:400 PNGase F stock to each sample and topped off with 100 mL of 20 mM NaHCO_3,_ this was incubated at 37 °C overnight. The released glycans were collected in a 2 mL Eppendorf by washing the gel pieces with 3 x 200 mL of water, 200 mL of acetonitrile (MeCN), 200 mL of water, and finally 200 mL of MeCN. The released glycans were dried, 20 mL of 1% formic acid was added to each sample, and the samples were incubated at room temperature for 40 min and then redried. 5 mL of 2-AB labelling solution was added to each sample and incubated for 30 min at 65 °C followed by shaking of the samples by hand and incubated for another 1.5 hr at 65 °C. Excess 2-AB was removed using Whatmann 3MM paper and washed with 1.5 mL MeCN (x7) and vortexed for 15 min. Lastly, 1.8 mL of water was used to wash the *N*-glycans that were dried and ready for UPLC analysis. The 2-aminobenzamide (2-AB) label was prepared as follows; NaB_3_CN (483.84 mg, 7 mmol) was dissolved in 5.376 mL of DMSO. 2-AB (368.64 mg, 2 mmol) was dissolved in 2.307 mL acetic acid. The two solutions were mixed gently over ice.

### IgG affinity purification, *N*-glycan release and labelling (AQC) protocol from human serum (Irish cohort)

The following buffers were prepared; 1% NaCl (washing buffer, 0.1% NaN_3_), 0.2 M glycine (elution buffer, at pH 2.5, 0.1% NaN_3_), 270 mM Phosphate-buffered saline (PBS binding buffer, at pH 7.4, 0.1% NaN_3_), 1 M Tris–HCl (neutralisation buffer, at pH 9, 0.1% NaN_3_). All sera samples in the pilot study (*n* = 12) were affinity purified on a Hamilton Starlet Liquid Handling Station in a 96-well plate format according to the literature report (O'Flaherty et al. 2019). In short, IgG glycoproteins were extracted from the serum of each participant in the Irish cohort (*n* = 12, 50 mL per well), the human IgG standards (50 mL, *n* = 3) and normal human serum (NHS, 50 mL, *n* = 3) and from blank wells (50 mL PBS, *n* = 3) using Protein G Phynexus Phytips. All samples were processed in tandem in a 96 well plate. The Phytips were pre-equilibrated (200 mL per well, 270 mM PBS, pH 7.4, 1 g/L NaN_3_, 3 cycles, 5 mL/s). Following this, affinity purification was performed to capture the IgG on the Phytip (50 mL human serum per well, 170 mL mixing volume, 20 cycles, 20 mL/s).The samples captured on the Phytip were then washed with PBS binding buffer (170 mL per well mixing volume, 270 mM PBS, pH 7.4, 1 g/L NaN_3_, 9 cycles, 5 mL/s), following this all samples were washed with 1% NaCl (0.1% NaN_3_, 3 cycles, 5 mL/s). The purified IgG was eluted into three separate Greiner plates (3 x 80 mL per well, 0.2 M Glycine buffer, pH 2.5, 3 cycles each, 4 mL/s). Neutralisation buffer (10 mL per well, 1 M Tris–HCl buffer, pH 9.0) was added to the first and second Greiner plates containing the samples and they were pooled together (resulting solution ~160 μL). Protein concentration was determined by UV–Vis spectroscopy at 290 nm. The absorbance values were correlated with a standard curve generated from known protein concentrations. The IgG protein concentration of the cystinosis cohort (*n* = 12) ranged from 0.61–1.34 μg/μL (mean ± std, 0.98 ± 0.20 μg/μL). Denaturation, alkylation, and enzymatic release of IgG *N*-glycans was performed according to the literature protocol (O'Flaherty et al. 2019) except for the following: *N*-glycans were released manually using 10KDa Nanosep cartridges, 0.5 M IAA was used for the alkylation, PNGase F treatment (0.416 mL, 25 mM SBC, final concentration 208 U/mL) was performed at 37 °C for 1 hr followed by fluorescent labelling. A 40 μL volume of collected *N*-glycans was reacted with 100 μL aminoquinolyl-*N*-hydroxysuccinimidyl carbamate (AQC) (3 mg/mL) in MeCN. Excess label was cleaned using a HyperSep-96Dial plate 100 mg Well Plate and 95% MeCN, and the *N*-glycans were collected in 20% MeCN. The final sample was dried down.

### IgG affinity purification, *N*-glycan release and labelling (AQC) protocol for human plasma (Boston cohort)

*N*-Glycan analysis was performed as described previously using a Hamilton Starlet Liquid Handling Station (Stöckmann et al. 2015; Cheng et al. 2020). IgG was purified from plasma samples using Protein G plates and re-suspended into 384-well ultrafiltration plates. Denaturation buffer (100 mM SBC, 50 mM dithiothreitol (DTT), 0.1% sodium dodecyl sulfate (SDS) was then added into 384 well plates. After 10 min at room temperature, the plates were incubated at 95 °C for 10 min and then equilibrated back to room temperature for 10 min. Following equilibration, 1 M IAA was dispensed into each well followed by deglycosylation reagents (PNGase F (0.416 mL, 25 mM SBC, final concentration 208 U/mL). Plates were then incubated on a shaker for 30 min at 38 °C and attached onto a collection plate and centrifuged. A solution of 25 mM SBC was then dispensed into each well, and the plate assembly was centrifuged to collect the flow through. A 5 μl volume of collected glycan sample was mixed with 11.6 μl aminoquinolyl-*N*-hydroxysuccinimidyl carbamate (AQC) (3 mg/mL) in MeCN and transferred to the UPLC system. Note that the Boston cohort and Irish cohort (Tables S1.4 and S1.2) were analysed at different time points.

### Ultra performance liquid chromatography (UPLC) for *N*-glycan visualisation and quantification

UPLC was performed as per literature report (Stöckmann et al. 2015) and GU values were compared to those reported in (O'Flaherty et al. 2019). In short, *N*-glycans was visualised and quantified using UPLC and fluorescence detection on a Waters ACQUITY UPLC H-Class instrument consisting of a binary solvent manager, sample manager, and fluorescence detector using the control of Empower 3 software (Waters, Milford, MA). The HILIC separation were performed using a Waters Bridged Ethylene Hybrid (BEH) Glycan column (130 Å, 1.7 μm particle, 2.1 mm x 150 mm) with 50 mM ammonium formate (pH 4.4) as solvent A and MeCN as solvent B. The ACQUITY UPLC H-Class was fitted with a 0.2 μm filter. An injection volume of 19 μL was prepared in 70% v/v MeCN for each injection for the Irish Samples (for serum and IgG *N*-glycans). An injection volume of 15 μL was prepared in 70% v/v MeCN for each injection for the Boston Samples. Samples were maintained at 5 °C before injection, and the column was set at 40 °C for separation. The FLD excitation/emission wavelength were λem = 245 nm and λex =395 nm for AQC and λem = 420 nm and λex = 330 nm for 2AB. The system was calibrated using an external standard of 2-AB labelled dextran. A fifth-order polynomial distribution curve was fitted to the dextran ladder to assign glucose units (GU) values from retention times (using Empower software from Waters). The separation of IgG *N*-glycans labelled with AQC and for serum *N*-glycans labelled with 2-AB are as follows; a linear gradient of 70–30% MeCN (0.56 mL/min, 20 min) and a linear gradient of 70–30% MeCN (0.56 mL/min, 30 min). The system was calibrated using an external standard of 2-AB labelled dextran. A fifth-order polynomial distribution curve was fitted to the dextran ladder to assign glucose units (GU) values from retention times (using Empower software from Waters).The chromatograms obtained for serum *N*-glycans were integrated for 46 glycan peaks (GPs) and reported as percentages of total glycans (Table S1 (Saldova et al. 2014)). For IgG *N*-glycans were integrated for 23 GPs reported as a percentage of total glycans (Table S2 and S3 (Stöckmann et al. 2015, Cheng et al. 2016)). The Boston Cohort (used as a control for IgG *N*-glycan analysis), IgG *N*-glycan peaks were integrated for 23 GPs (Table S4 (Cheng et al. 2020))

### Statistical analysis

Principal components analyses (PCA) was carried out with the get_pca function of glycowork (Thomès et al. 2021) (version 1.2.0) and uniform manifold approximation and projection (UMAP) with the umap-learn package (version 0.5.4) using the Irish (serum and IgG *N*-glycan data) and Boston cohorts (IgG *N*-glycan data), with the respective default parameters of PCA and UMAP. For each PCA and UMAP, biplots were produced of the first two principal components/dimensions. Further, unsupervised hierarchical clustering of the Irish cohort was performed with the get_heatmap function of glycowork, using their GP1-GP23 values, derived glycan traits, and IgG titres. For the Irish cohort, the serum and IgG *N*-glycans were analysed. For each response variable (glycan peaks, traits, and IgG titres), linear models were fitted assuming a normal distribution for errors, including the effects of age (linear), sex, and NC status. Sequential ANOVA (type-I sums of squares) was carried out and performed F-tests to assess the significance of the effects. Simultaneously, hierarchical clustering (HC) was performed for the individuals in the Irish cohort using only the glycan peak (this refers to an integrated peak on the UPLC chromatogram that can contain one or more *N*-glycan species (Zhao et al. 2018, Liu and Liu 2021, Xie et al. 2021)) variables. The Euclidean distance and Ward’s clustering methods were used. Model-based clustering was performed with the same data, assuming a mixture of two Gaussian distributions. R (version 4.3.3) package mclust was used to carry out these analyses (Scrucca et al. 2016). For the Boston cohort data, the effect of age on the mean and variance of GPs was assumed by stratifying the age into three groups: younger than 5, between 5 (inclusive) and 10 yr old, and older than 10 (inclusive) (plasma IgG *N*-glycans). Normal models were fitted with the effects of age in the mean and variance (to account for variance heterogeneity) and carried out likelihood-ratio tests for nested models to assess the significance of the age and sex effects. R (version 4.3.3) package gamlss (Stasinopoulos and Rigby 2007) was used to fit these models (R Core Team 2014). The biplots from the principal components were analysed and generated using package (Vince Vu 2011); all other visualisations were produced using package ggplot2 (Wickham 2016).

## Supplementary Material

NC_Supporting_(S1-S4)_cwaf047

NC_Supporting_Figures_(S1-S4)_cwaf047

NC_Supporting_Tables_(S1-S7)_cwaf047

## Data Availability

Where possible, all generated data is included in the Supporting Informations. Some personal data is unavailable due to privacy restrictions.
