# Supplementary material for: Serum N-glycosylation is altered in Nephropathic Cystinosis
Source: Glycobiology. 2025 Aug 22;35(10):cwaf047. doi: 10.1093/glycob/cwaf047 (PMC12406699; doi:10.1093/glycob/cwaf047)
Supplement: NC_Supporting_Figures_(S1-S4)_cwaf047 [file nc_supporting_figures_(s1-s4)_cwaf047.pdf]

SUPPORTING FIGURES (S1-S4)

Serum *N*-Glycosylation is Altered in Nephropathic Cystinosis

Andreea Cislaru[1], Radka Saldova[2,3,4], Alessandra Heggenstaller[5], Peter A. Nigrovic[6,7], Emily Harlin[1], Gordon Greville[8], Rafael De Andrade Moral[9], Daniel Bojar[10], Atif Awan[5], Róisín O'Flaherty[1,4,11] *

[1]Department of Chemistry, Maynooth University, Maynooth, Co. Kildare, Ireland, W23 F2H6.

[2]GlycoScience Group, National Institute for Bioprocessing Research and Training (NIBRT), Fosters Avenue, Mount Merrion, Blackrock, Co. Dublin, Ireland, A94X099.

[3]UCD School of Medicine, College of Health and Agricultural Science (CHAS), University College Dublin (UCD), Dublin, Ireland.

[4]CÚRAM, Science Foundation Ireland Research Centre for Medical Devices, Biomedical Sciences, University of Galway, Co. Galway, Ireland. H91 W2TY.

[5]Children's Health Ireland (CHI) at Temple Street, Temple Street, Dublin 1, Ireland, D01 XD99.

[6]Division of Immunology, Boston Children's Hospital, Harvard Medical School, Boston, MA, USA, 02115.

[7]Division of Rheumatology, Inflammation, and Immunity, Brigham and Women's Hospital, Harvard Medical School, Boston, MA, USA, 02115.

[8]Department of Biology, Maynooth University, Maynooth, Co. Kildare, Ireland, W23 F2H6.

[9]Department of Mathematics and Statistics, Maynooth University, Maynooth, Co. Kildare, Ireland, W23 F2H6.

[10]Wallenberg Centre for Molecular and Translational Medicine, Department of Chemistry and Molecular Biology, University of Gothenburg, 41390 Gothenburg, Sweden.

[11]Kathleen Lonsdale Institute for Human Health Research, Maynooth University, Maynooth, Co. Kildare, Ireland, W23 F2H6.

*Correspondence: +353 1 708 6477, *roisin.oflahery@mu.ie

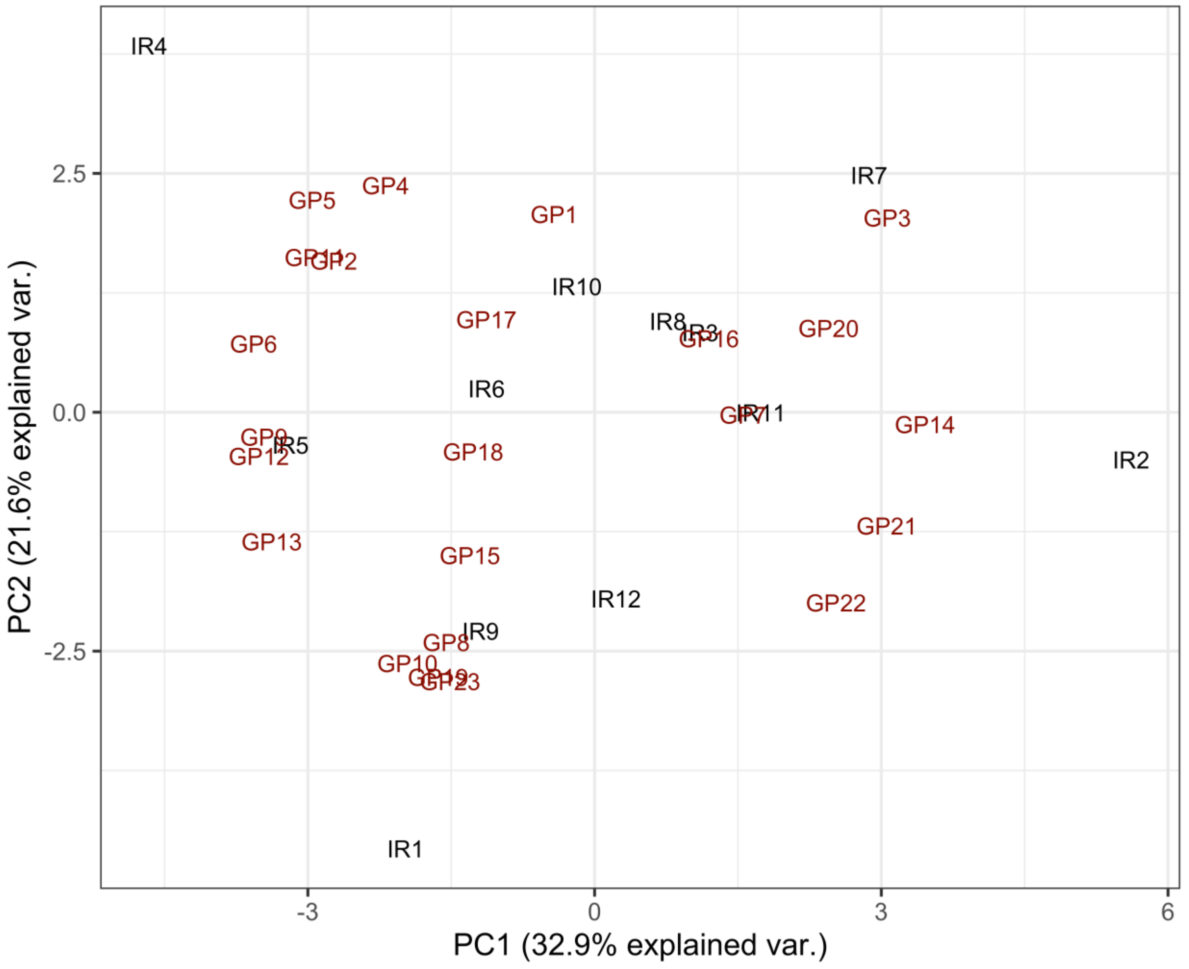

Figure S1. Principle component analysis (PCA) of Irish cohort participants using their IgG *N*-glycans with 23 GPs and IgG titres for the unknown juveniles with/without NC (n=12, age 2-14, males and females).

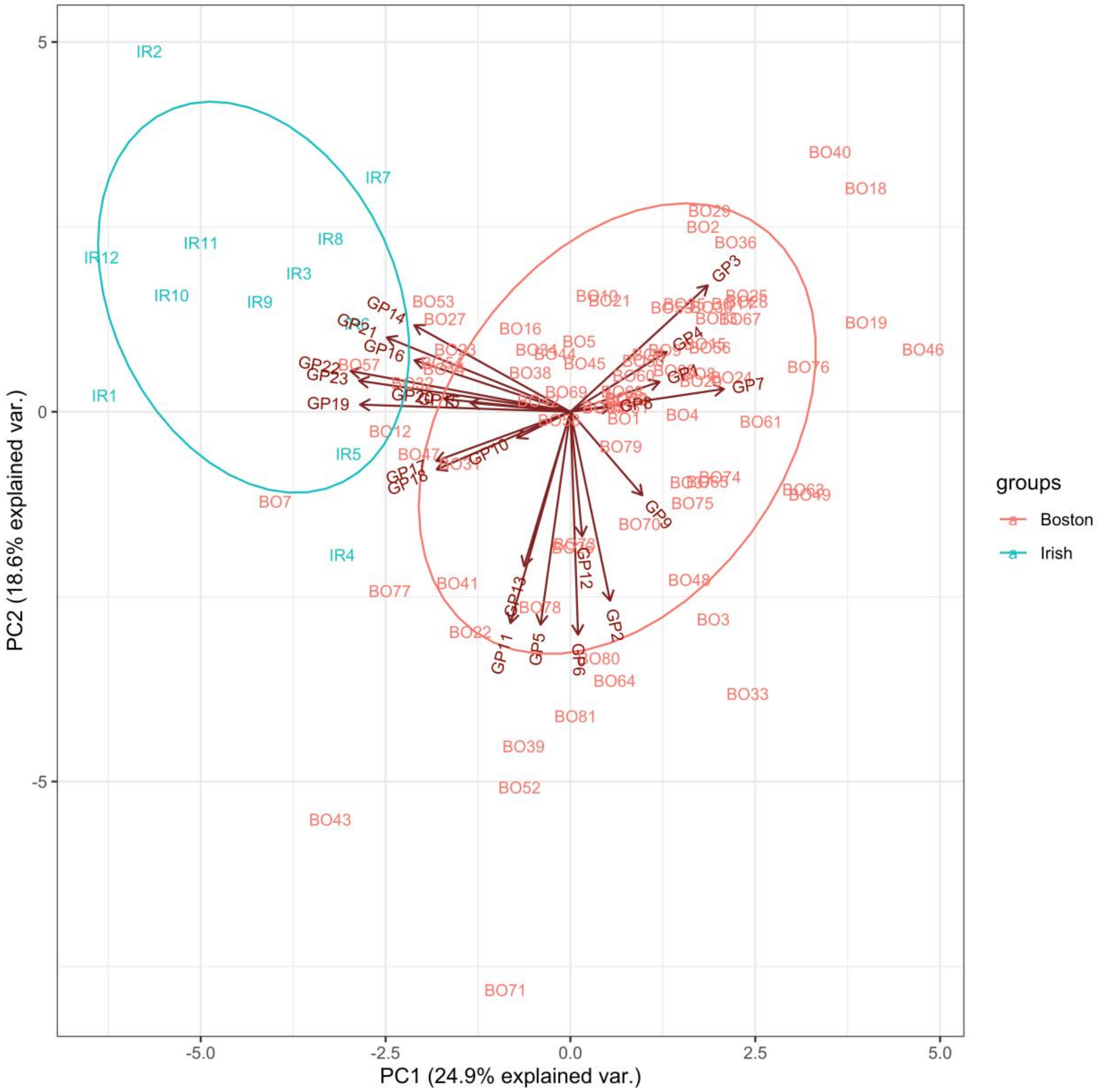

Figure S2. Principle component analysis (PCA) for IgG *N*-glycosylation of Irish cohort (n=12, age 2-14 years, males and females) and the control Boston cohort (n=81, age 2-14 years, males and females, previously described (Cheng, H.D., Tirosh, I., et al. 2020)) were analysed using GP1-23.

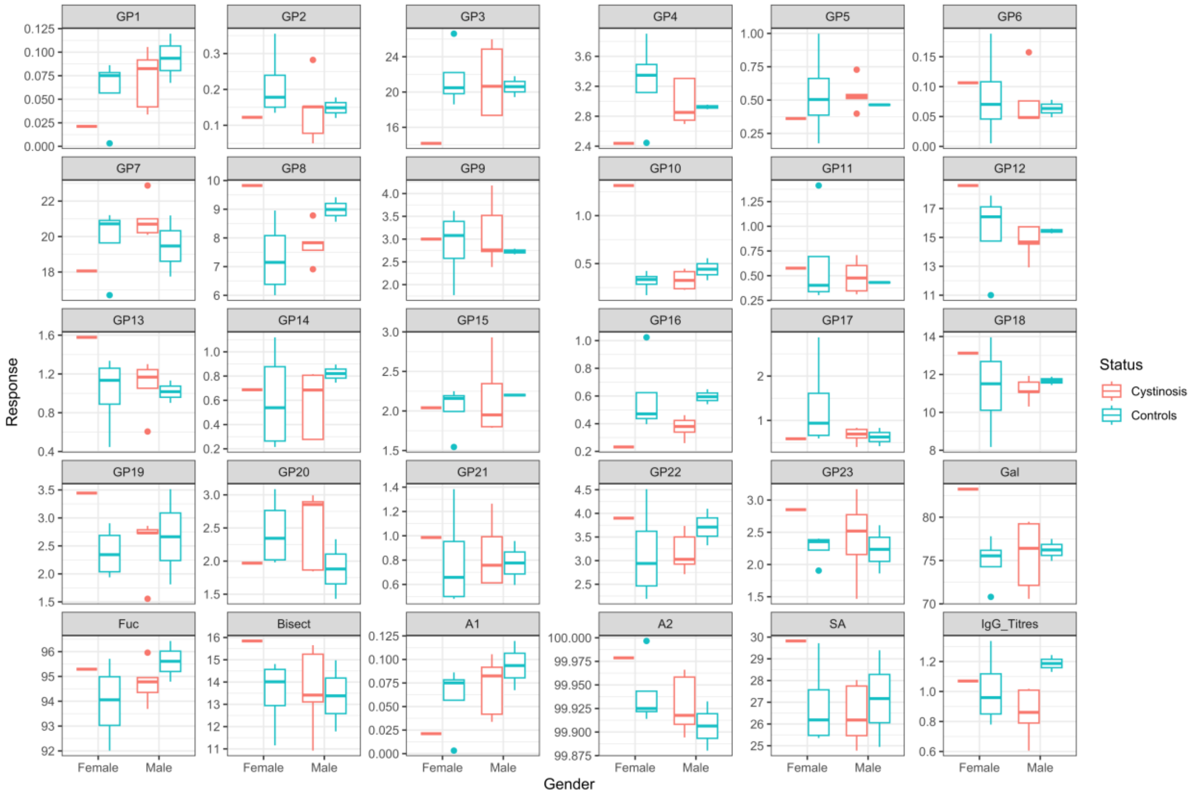

Figure S3. Boxplots of Irish cohort participants using their 23 IgG *N*-glycan GPs, glycan traits, IgG titres for the unblinded juveniles with/without NC (n=12, age 2-14, males and females).

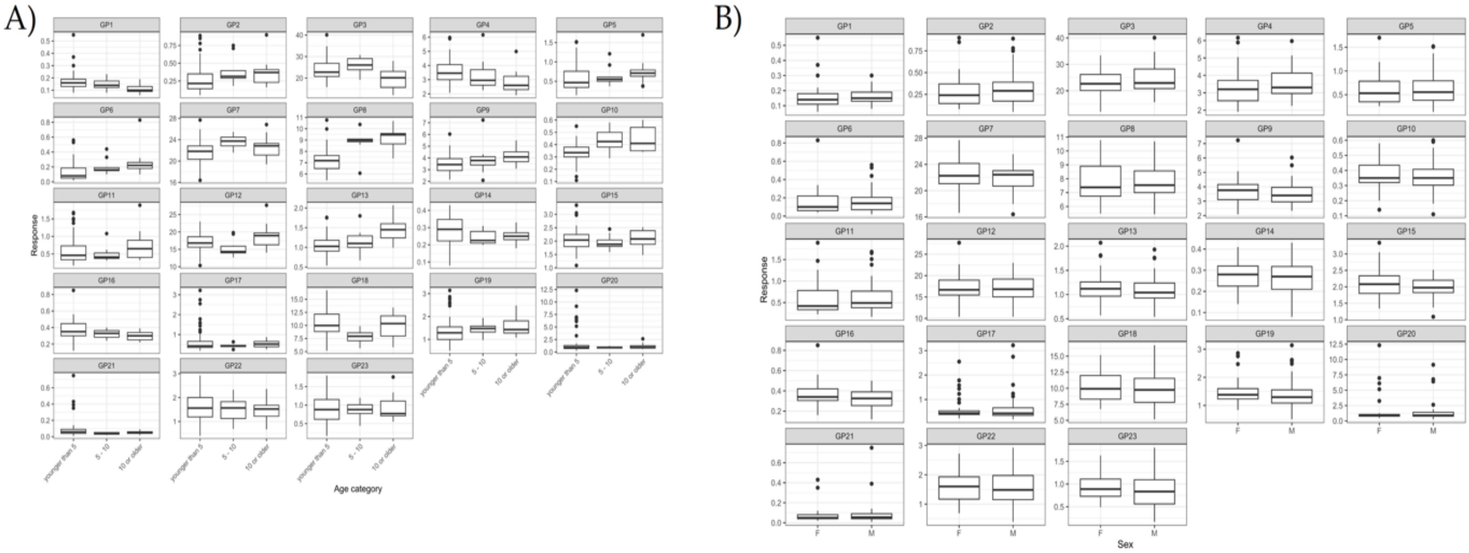

Figure S4. Boston control cohort (n=81, males and females) discrimination for age and sex: A) Boxplot analysis of 23 GPs for three age groups (younger than 5 years, between 5 and 10 years, and 10-14 years). B) Boxplot analysis of 23 GPs for females (F) compared to males (M).

References

Cheng HD, Tirosh I, de Haan N, Stöckmann H, Adamczyk B, McManus CA, O'Flaherty R, Greville G, Saldova R, Bonilla FA*, et al.* 2020. IgG Fc glycosylation as an axis of humoral immunity in childhood. *J Allergy Clin Immunol*, 145:710-713.e719.
